# *Bacillus megaterium* favours CO₂ mineralization into CaCO₃ over the ureolytic pathway

**DOI:** 10.1038/s41598-025-07323-9

**Published:** 2025-07-01

**Authors:** Margherita Cappa, Camilla Perego, Dimitrios Terzis, Pamela Principi

**Affiliations:** 1 Environmental Biotechnology, Institute of Microbiology, Department of Environment, Construction and Design, University of Applied Sciences and Arts of Southern Switzerland (SUPSI), Manno, Switzerland; 2 Swiss Federal Institute of Technology, Lausanne (EPFL), Faculty of Environment, Architecture and Civil Engineering (ENAC), Lausanne, Switzerland; 3 Medusoil SA, EPFL Innovation Park Building A, 1015, Lausanne, Switzerland

**Keywords:** Biomaterials, Biogeochemistry, Engineering

## Abstract

Microbially induced calcite precipitation (MICP) has long been the focus of material scientists, environmental microbiologists and civil engineers because of its potential to yield biosynthesized binders that can serve as alternatives to cement or resins. Several microbial strains play crucial roles in this process and catalyse pathways for the formation of minerals, which are believed to substantially reduce the environmental impact of building materials and activities. Among the studied strains, *Bacillus megaterium* is not as common as *Sporosarcina* species. The latter microorganisms are well known to drive the fastest ureolytic-driven MICP process, i.e., precipitation of CaCO_3_ after urea breakdown into carbonate and CaCl_2_ addition to the system. This paper sheds light on the activities of *B. megaterium*, which possesses dual enzymatic capabilities for MICP and is equipped with both the enzymes urease and carbonic anhydrase. We postulate that, depending on the growth conditions, *B. megaterium* can activate either of these genes to ultimately induce CaCO_3_ precipitation. Herein, experiments are carried out in open and closed systems. C^13^-labelled urea is employed to identify the carbon source in the precipitated CaCO_3_. The results from Fourier transform infrared spectroscopy (FTIR) revealed the precipitation of calcite. In the presence of urea and CO_2_ at atmospheric levels, *B. megaterium* activates the ureolytic pathway to perform urea hydrolysis. However, at increased CO_2_ levels, more precisely, at levels greater than 470 times the atmospheric level, carbonic anhydrase is activated, catalysing the hydration of the molecule to produce HCO_3_^−^. When C^13^-labelled urea was utilized, only 6% of the precipitated CaCO_3_ mineral was linked to ureolysis, and it was found that the remaining 94% was formed due to the mineralization of CO_2_. Overall, in this work, we aim to introduce the process conditions and protocols that favour the sequestration of atmospheric CO_2_ as CaCO_3_ via the metabolic activities of *B. megaterium*.

**Supplementary Information:**

The online version contains supplementary material available at 10.1038/s41598-025-07323-9.

## Introduction

In this work, we aim to investigate a less explored microorganism in the traditional field of microbially induced calcite precipitation (MICP), which is dominated by ureolytic strains^1–4^. To understand the motivation behind this work, one should consider that the traditional MICP pathway involves the breakdown of urea (an organic fertilizer produced via the combination of liquid CO_2_ and liquid ammonia) by urease-producing microorganisms such as *Sporosarcina spp.* into CO_3_^2−^ and NH_4_^+^ (Eq. 1). The ammonium ion is an unwanted byproduct that must be completely removed as a trade-off in the otherwise beneficial MICP process, in which CaCO_3_ precipitate acts as a binder for building applications, commonly referred to as biocement (Eq. 2)^5^.

1 $$\text{CH}_4\text{N}_2\text{O} + 2\text{H}_2\text{O} \to 2\text{NH}_4{}^{+}\: + \text{CO}_3{}^{{2-}}$$

2 $$\text{Ca}^{2+}\:+\:\text{CO}_3{}^{2-} \leftrightarrow \text{CaCO}_3$$

In addition to traditional ureolytic MICP, among the different metabolic pathways that facilitate carbonate precipitation, the use of carbonic anhydrase (CA) is attracting interest. In recent works, microbes with genetic information for the CA enzyme have been mobilized in an attempt to eliminate ammonium, for example, in the work of^6^, in which *Bacillus licheniformis* was used in bioaugmentation protocols to improve clay structure. In this method, the applied MICP pathway emerged from a preliminary step in which hydrated aqueous CO_2_ formed H_2_CO_3_ upon reaction with water and subsequently ionized, yielding HCO_3_^−^ and water (Eq. 3). The newly ionized carbonate anion then combined with free Ca^2+^ cations to form CaCO_3_ in one of its metastable phases, i.e., into the amorphous calcium carbonates (ACCs) vaterite or aragonite or to directly form calcite (Eq. 2). CA is known to serve as a nucleation site in this process^6,7^.

3 $$2\text{HCO}_3{}^{-}\:+\:2\text{OH}^{-}\:\leftrightarrow\: 2\text{CO}_3{}^{2-}\:+\: 2\text{H}_2\text{O}$$

**Fig. 1:**
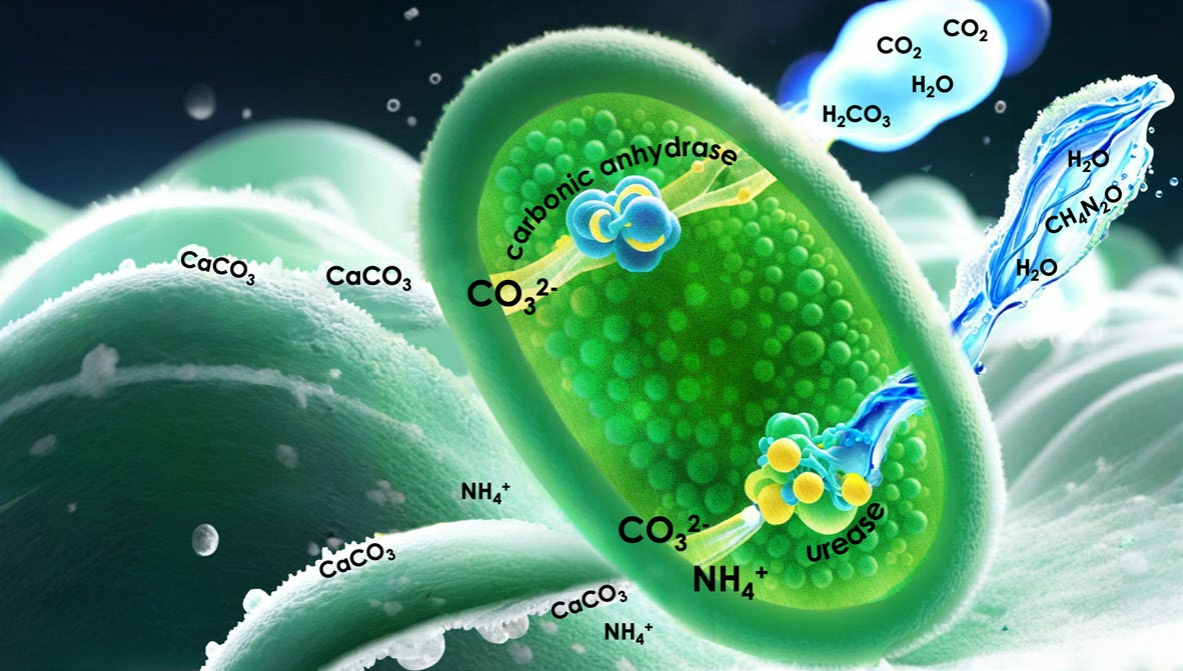
Illustration of urease and CA-induced MICP in B. megaterium using urea and CO_2_ as reactants, respectively.

We therefore focused on *Bacillus megaterium*, a species with genetic determinants for both the enzymes urease and carbonic anhydrase. These two pathways, in fact, can be activated at the same time synergistically^8^, but it is unclear whether selective activation can occur. Mineralization of gaseous CO_2_ by *B. megaterium* has been reported in^9^, and given increasing interest in the direct use of atmospheric CO_2_ in industrial processes, in this study, we suggest a framework for tracing the carbon source of precipitated CaCO_3_ and quantifying the fraction that originates from CO_2_ (Fig. 1).

## Materials and methods

*Bacillus megaterium* is an aerobic gram-positive bacterium commonly present in soils^10^. It received its unique name from its dimensions: 4 μm long and diameter of 1.5 μm^11^. The interest in this taxon lies in its versatility; it has been applied for recombinant protein production^12^, soil bioremediation^13^, and microbially induced precipitation^14^. The enzyme urease catalyses the reaction, and one mole of urea is hydrolysed to ammonia and carbonic acid^15^. This leads to an increase in pH and the number of negative ions on the cell surface, which attract positively charged calcium ions, leading to CaCO_3_ precipitation. *B. megaterium* has the ability to exploit another pathway to induce carbonate precipitation^10^ via CA-mediated precipitation. An alkaline environment induces a reaction between calcium ions and HCO_3_^−^ and ultimately leads to calcium carbonate precipitation. Nevertheless, environmental conditions, such as pH, temperature^16^ and the presence of ions like magnesium^17,18^, have a complex and significant effect on the precipitation of CaCO₃ polymorphs, influencing not only the crystallization process but also the morphology and stability of the resulting phases. Despite this, the selective activation of ureolytic metabolism as a mechanism for controlling CaCO₃ polymorphism remains less explored, warranting further investigation to better understand its role in microbial-induced calcium carbonate precipitation.

### CaCO_3_ precipitation via the urease pathway

To confirm the urea hydrolysis pathway in MICP, a first round of growth experiments (U test) was performed in aerobic 100 mL flasks on the basis of conditions proposed by^19^. *B. megaterium* (DSM 32) was grown in 15 g/L yeast extract, 10 g/L peptone, and 10 g/L NaCl (growth medium) for 24 h. All the media components were obtained from Sigma Aldrich unless otherwise indicated. Subsequently, 4.5 mL of *B. megaterium* culture was inoculated (OD_600_ = 0.7) in 30 mL of media supplemented with urea and CaCl_2_. Both components were filtered through 0.22 μm filters (Sigma‒Aldrich) and added in equimolar proportions at 1.5 M. The flasks were incubated in triplicate for four days at 30 °C and 100 rpm. Daily feedings of the growth medium were performed, and pH was monitored at the end of each experiment together with precipitate yield, absorbance and electrical conductivity (see Supplementary Figure S1). One should consider herein that alkaline environments can be linked to the presence of peptone itself (10 g/L), which may influence the pH of the medium, or potentially magnesium, which is known to preferentially precipitate aragonite^20^. However, magnesium was not used in this study. Furthermore, abiotic control samples, including peptone, were tested in this work and yielded no precipitation, supporting the notion that the presence of peptone alone does not significantly impact the absorption or hydration of CO₂ in the culture medium. Data from these abiotic tests are provided in the Supplementary Figure S1. In CO₂-infused media, suboptimal pH conditions for CaCO₃ precipitation are expected due to acidification. While such environments are often considered unfavorable for biomineralization, recent studies show that *B. megaterium* retains urease activity and supports calcite formation even below neutral pH. More precisely^21^ observed measurable growth and precipitation at pH 6–7, and Manna et al. (2024)^22^ confirmed these findings across a broader acidic-to-alkaline range. These results indicate that CaCO_3_ precipitation cannot be excluded under mildly acidic, CO₂-rich conditions. Additionally, we postulate that peptone concentrations around 10 g/L are typically sufficient to support bacterial growth and promote ammonia production from urea (which was supplied at 90 g/L), thus contributing to medium alkalinization, as described by^23^. These starting hypotheses highlight how these factors—alkaline environment, peptone, and the absence of magnesium—contribute to the formation of CaCO₃ polymorphs via enzymatic pathways in the proposed experimental system.

### CaCO_3_ precipitation via the carbonic anhydrase pathway

The second round of experiments (CA test) was designed to evaluate CaCO_3_ formation via the carbonic anhydrase pathway. For this purpose, 2.5 mL of *B. megaterium* grown for 24 h was inoculated (OD_600_ = 0.7) in fresh medium supplemented with C^13^-labelled urea (Sigma‒Aldrich, CAS 58069-82-2), and CaCl_2_ was added in an equimolar amount at 1.5 M (Table 1). This experiment was conducted in closed vials in triplicate. Thirty millilitres of CO_2,_ corresponding to 1.24 × 10^−3^, was injected into each vial via a 30 mL syringe. The samples were incubated for four days at 30 °C and 100 rpm. Daily feedings of the growth medium were performed, and pH was corrected to above a value of 8. The precipitate in this test was used to determine the origins of the carbon in the formed CaCO_3_.

**Table 1:** Treatment protocols and test conditions.

Test name	Growth Medium	Urea and CaCl_2_	CO_2_ available in liquid phase	Environment
U Test	Yeast extract 15 g/L Peptone 10 g/L NaCl 10 g/L	1.5 M	5.44 × 10^−7^	Open flasks
CA Test			2.55 × 10^−4^	Closed vials

The available CO_2_ in the liquid phase was calculated on the basis of Henry’s law considering Henry’s law constants and the gas partial pressure^24^.

### Precipitate analysis

The collected samples were centrifuged for 4 min at 624 × g, and the pellet was washed with acetone (Sigma Aldrich, 99% purity). After the pellet was dried overnight at 60 °C, a second washing step with 96% ethanol was performed. The samples were subsequently dried and heated at 450 °C for two hours before Fourier transform infrared (FTIR) spectroscopy (Spectrum two, Perkin Elmer) and scanning electron microscopy (SEM) analyses with energy dispersive X-ray spectroscopy (EDX) (Phenom XL, Thermo Scientific) were performed. The organic and inorganic origins of the CaCO_3_ precipitate was investigated by analysing the C^13^ -labelled fraction of carbonate via the double-capsule technique described in^20^.

## Results

### CaCO_3_ production in the U test

**Fig. 2:**
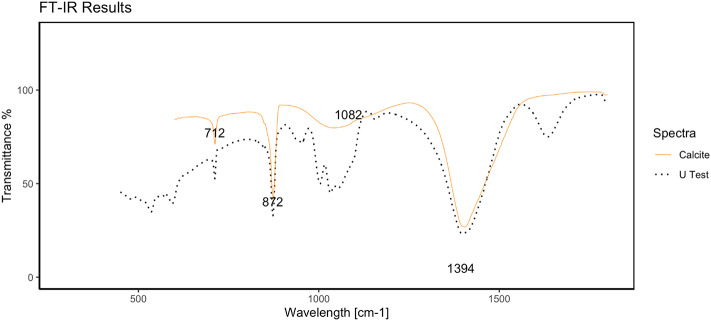
U FTIR spectra of the calcium carbonate precipitate peaks compared with those of pure calcium carbonate peaks.

The FTIR spectra of the precipitates from the U test are reported in Fig. 2. The yellow line represents the calcite spectrum acquired from a reference database^25^. The wavelength range between 400 and 1500 nm contains characteristic peaks for calcite: the absorptions at 712 cm^−1^, 872 cm^−1^, 1082 cm^−1^, and 1394 cm^−1^ are influenced by the different ways in which C-O bonds form carbonate^26^. The black dotted line corresponds to the precipitate sample from the U test, and as expected, the characteristic peaks at 712 cm^−1^, 872 cm^−1^, and 1394 cm^−1^ are identified and in agreement with the reference spectrum. Additionally, in the spectrum at approximately 1082 cm^−1^, it is possible to distinguish a wider peak that spans several peaks. We postulate that this is due to the trace amount of other inorganic material that was still present in the pellet despite the washing procedure.

### CaCO_3_ production in the CA test

**Fig. 3:**
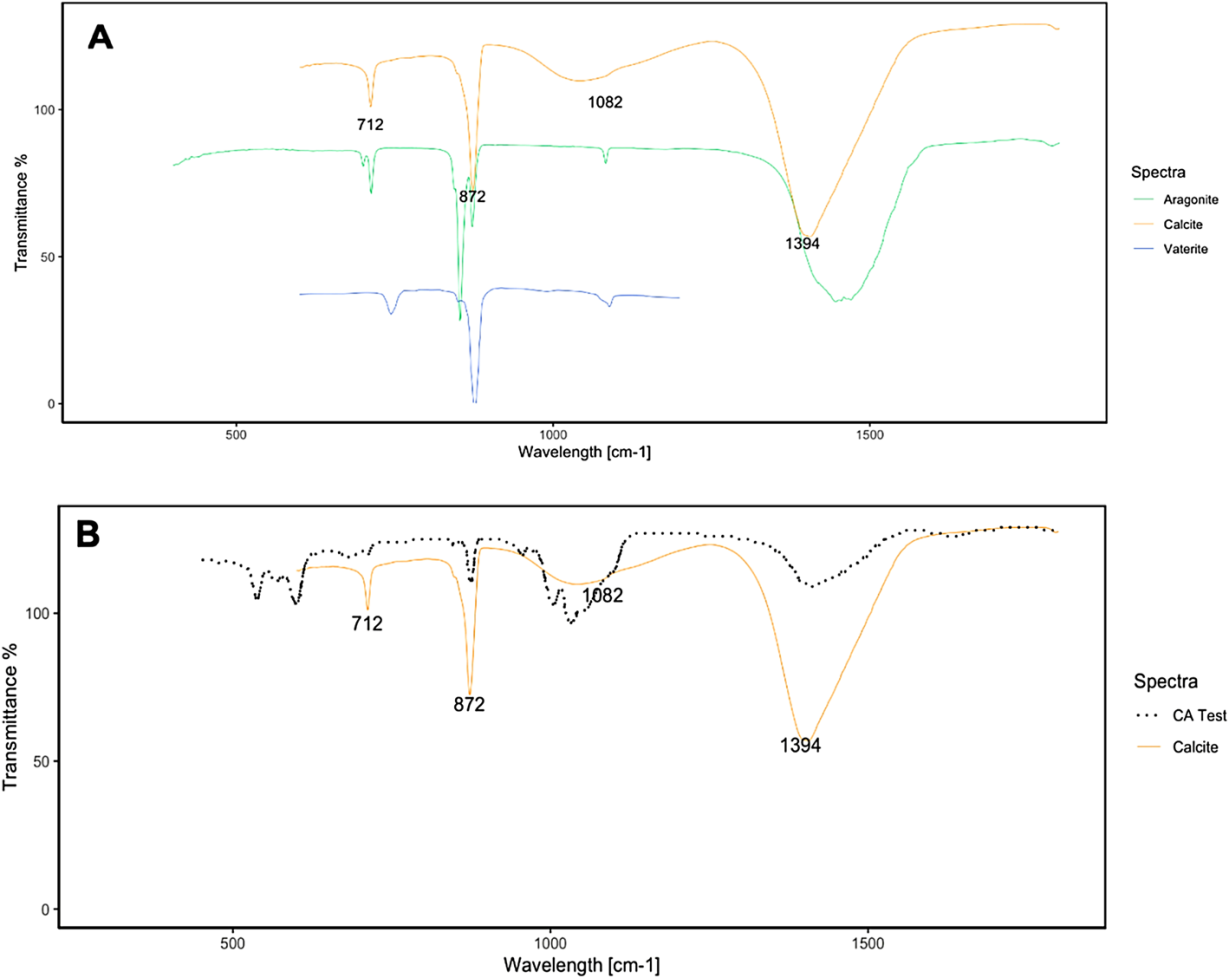
**(A)** FTIR spectra of calcite, aragonite and vaterite; **(B)** CA test results compared with those of calcite.

The FTIR spectra of carbonate from CA test are reported in Figure 3B. Similar to that in the previous experiment, the precipitate was identified as CaCO_3_ by EDX (see Supplementary Figure, S1). To elucidate the polymorphic phase of CaCO_3_, the spectra were subsequently analysed and compared: calcite and aragonite data were collected from the reference database (Perkin Elmer Spectrum IR), whereas the spectra of vaterite were taken from the work of^27^ (Figure 3A). As mentioned above, calcium carbonate has three characteristic IR absorption peaks in the range between 400 cm ^and 1^ and 1500 cm^−1^ due to the interaction of the C-O bonds with infrared radiation. CaCO_3_, in fact, has three crystalline structures, all with the same chemical composition: calcite, aragonite, vaterite, and each crystalline structure can be associated with a corresponding spectrum (Figure 3A). A comparison of the calcite and aragonite FTIR spectra (Figure 3A) revealed that in the aragonite absorption spectra, the peak at 712 cm^−1^ is shifted, with an additional peak at 700 cm^−1^; this peak is not present in the calcite phase. In both the vaterite and calcite spectra (Figure 3A), the peak at 875 cm^−1^ is matched; on the other hand, the peak at 712 cm^−1^ present in calcite is shifted to 748 cm^−1^ for vaterite^28^. Furthermore, the third characteristic peak of calcite (at 1082 cm^−1^) is slightly shifted in the vaterite sample. In the CA test, the obtained spectra are consistent with the peaks corresponding to calcite (Figure 3A), which confirms that *B. megaterium*-induced MICP leads to the formation of calcite^27^.

**Fig. 4:**
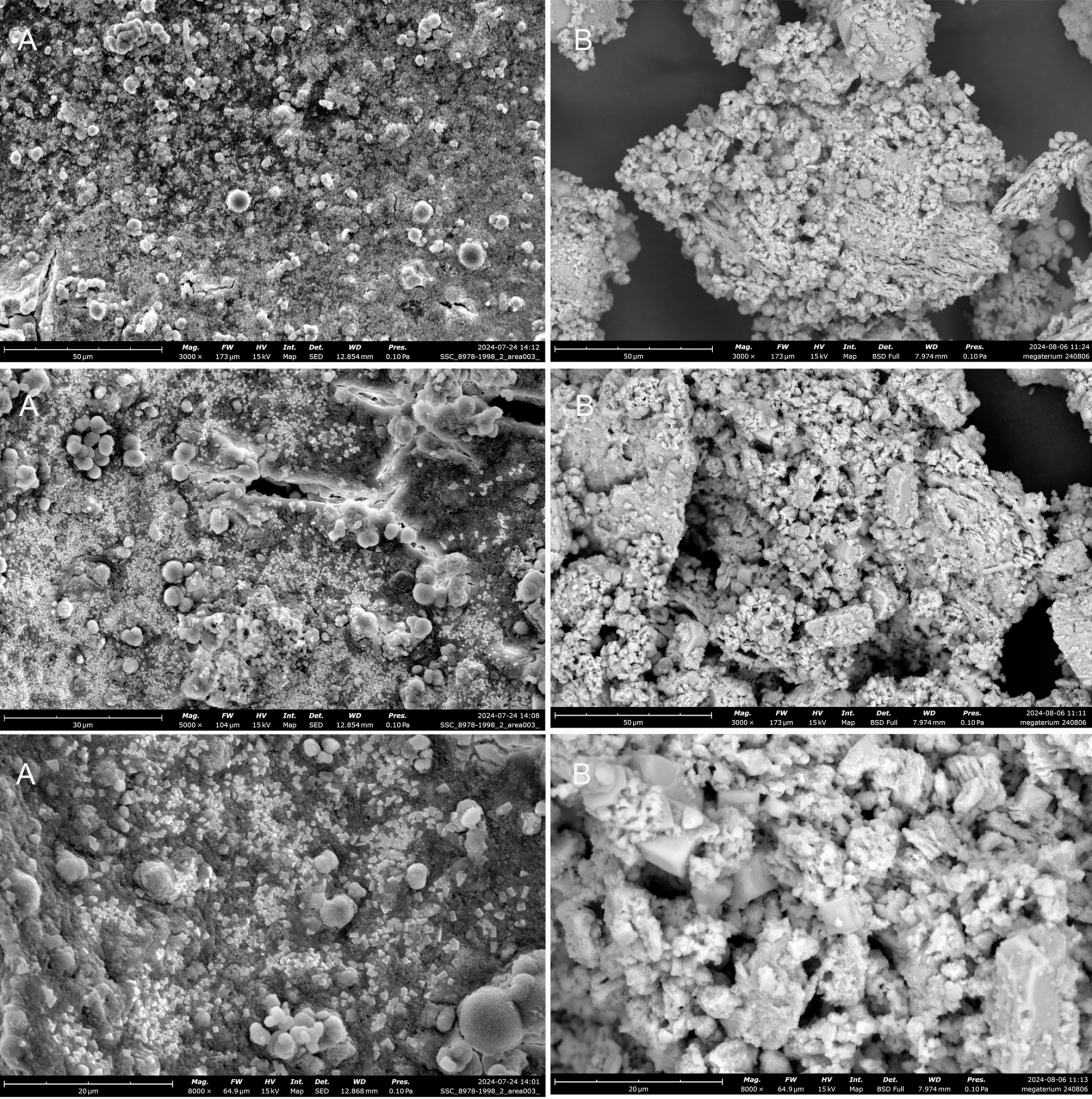
CA test scanning electron microscopic observations at magnifications of 3000x, 5000x, 8000x at 15 kV and 10 Pa; **(A)** Pellet collected from fresh samples; **(B)** pellet postdrying and postwashing.

In addition to FTIR analysis, using the pellet collected from the CA Test, the textural and morphological characteristics of the crystals formed by *B. megaterium* were investigated using SEM. In the samples collected from the fresh medium (Figure 4A) and from the double-washed, dried pellet (Figure 4B), no dominant morphological or textural features were identified, and *B. megaterium* cells could not be distinguished. This could be explained by the predominant distribution of the microorganisms in the supernatant rather than in the pellet collected from the bottom of the flask after centrifugation. In the washed and dried precipitate (Figure 4B), as expected, no microbial cells were identified, but a clearer and more hierarchical matrix was observed for the residual crystals that resisted the washing and drying process. More precisely, the inorganic fraction had different morphological structures, i.e., rhombohedral, spherical, and amorphous. We postulate that the applied protocols resulted in the coexistence of various polymorphs of calcium carbonate, as observed in other MICP studies^14^. Therefore, it can be concluded that the final polymorph of calcium carbonate formed depends on the amount of dissolved organic carbon (DOC) present. When DOC is present in low quantities, calcite is more likely to form, whereas when DOC is present in high quantities, the formation of vaterite is favoured instead^8^.

The results from duplicate analyses using the double-capsule technique^20^ allowed for the quantification of the fraction of CaCO_3_ that contained the C^13^-labelled organic precursor (urea). Notably, 94.1% (mean value, with a standard deviation of ± 0.1%) of the total carbon was unlabelled, which implied that the vast majority of the precipitated CaCO_3_ was formed from the incorporation of inorganic carbon, i.e., CO_2_ was identified as the main carbon source and precursor of the mineral phase. This result highlights that in the presence of increased concentrations of CO_2_ (1.24 × 10^−3^), *B. megaterium* tends to exploit its carbonic anhydrase metabolic pathway predominantly instead of the ureolytic pathway for the synthesis of CaCO_3_.

## Conclusions

On the basis of the results presented in this work, it can be inferred that *Bacillus megaterium* (DSM 32) selectively activates urease- or anhydrase-driven metabolic pathways for the precipitation of CaCO_3_. The pathway depends on the conditions provided and medium composition. In the presence of urea, CaCl_2_ and CO_2_ at atmospheric levels, the urease pathway is favoured. This result was a fundamental starting point for subsequent experiments in which the growth conditions were altered by incorporating increased levels of CO_2_ into the medium. Under these conditions, the microorganisms activated the enzyme carbonic anhydrase, and the resulting minerals were linked to CO_2_ at a fraction of at least 94% of the total mineral yield. This inorganic origin of the carbon that was incorporated into CaCO_3_ leads to the conclusion that *B. megaterium* preferentially activates the carbonic anhydrase pathway under the provided conditions. Another important dimension of this finding, in addition to the obvious advantages of the direct sequestration of atmospheric CO_2_ into CaCO_3_, is the avoidance of potentially hazardous metabolic byproducts produced via the urease pathway, especially ammonia, which would require additional wastewater treatment steps. The findings of this work, along with the proposed protocol for the growth of *B. megaterium*, can have a significant impact on the future design of microbially induced carbon dioxide sequestration (MICO_2_S) strategies and the incorporation of CaCO_3_ as a binder mineral in building applications.

## Discussion

In this work, we used a known protocol^27^ for the growth and application of *Bacillus megaterium* for the precipitation of CaCO_3_ and benchmarked its efficiency using urea-driven MICP in open-flask experiments. The protocol was subsequently modified to allow for the incorporation of high concentrations of CO_2_ in a closed system, and we focused on the qualitative and quantitative analysis of the resulting precipitates. This was performed via textural observations via SEM and the use of the double-capsule technique^20^ to distinguish the origins of the carbon present (organic or inorganic) in the precipitate. In a closed system and under CO_2_ concentrations that exceeded 2300 times the concentration of CO_2_ in an open-air flask, i.e., atmospheric concentrations, the precipitated calcite was predominantly linked to CO2 mineralization, whereas urea labelled with C^13^ showed only a minor contribution (approximately 6%) to the resulting mineral as a precursor. These findings suggest a new protocol to potentially disassociate the ureolytic pathway with MICP and elucidates a new pathway for the potential valorisation of CO_2_ and its incorporation into mineral agents that can find multiple applications in real-world environments. The potential for bioremediation of surfaces in the built environment, including new buildings and monuments, presents promising opportunities, among others in heritage conservation. Microbial communities can self-inoculate, facilitating the consolidation and protection of stone materials^29^. Moreover, studies have demonstrated that the interaction between water availability and microbial communities significantly influences the degradation and conservation of stone monuments, supporting the broader application of this approach in bio-restoration practices^30,31^. By harnessing these processes, this methodology can contribute to sustainable conservation efforts and the mitigation of environmental damage to valuable structures. Overall, this study sets the foundation for understanding the kinetics of CO₂ mineralization and the relative contributions of different metabolic pathways, with future work potentially incorporating the use of enzymatic inhibitors or exploring the effects of varying CO₂ concentrations to further elucidate the mechanistic details of the process. As a proposed direction for future research, we recommend a detailed investigation of local pH dynamics and kinetic profiling of B. megaterium enzymatic activity under mildly acidic to neutral or alkaline conditions to better characterize its biomineralization dynamic and potential.

## Electronic supplementary material

Below is the link to the electronic supplementary material.

Supplementary Material 1

## Data Availability

The datasets used and/or analysed during the current study available from the corresponding author on reasonable request.
