# Supplementary material for: Bacillus megaterium favours CO₂ mineralization into CaCO₃ over the ureolytic pathway
Source: Sci Rep. 2025 Jul 1;15:21861. doi: 10.1038/s41598-025-07323-9 (PMC12216158; doi:10.1038/s41598-025-07323-9)
Supplement: Supplementary file 1 — Supplementary Material 1 [file 41598_2025_7323_MOESM1_ESM.docx]

Supplementary Figure S1


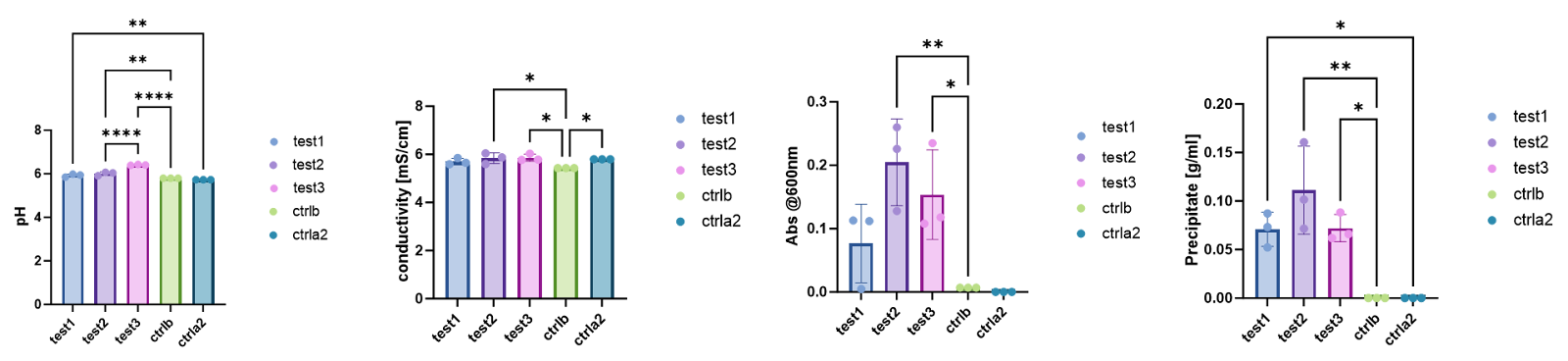


Figure S1 Monitoring of: pH, Electrical conductivity, Absorbance (600nm) and precipitate yield for triplicates (Test 1,2,3) and two control, abiotic samples (ctrla2,ctrlb)
